# Anterior-only stabilization using cage versus plating with bone autograft for the treatment of type II/IIA Hangman’s fracture combined with intervertebral disc injury

**DOI:** 10.1186/s13018-015-0164-1

**Published:** 2015-03-11

**Authors:** Fuxin Wei, Ximin Pan, Zhiyu Zhou, Shangbin Cui, Rui Zhong, Le Wang, Manman Gao, Ningning Chen, Zijian Liang, Xuenong Zou, Sheng Huang, Shaoyu Liu

**Affiliations:** Department of Spine Surgery, The First Affiliated Hospital and Orthopedic Research Institute of Sun Yat-sen University, Guangzhou, China; Department of Radiology, The First Affiliated Hospital of Sun Yat-sen University, Guangzhou, China; The Medical School of Shenzhen University, Shenzhen, China

**Keywords:** Hangman’s fracture, Treatment, Cage, Operation

## Abstract

**Background:**

Anterior C2/3 discectomy and interbody fusion (ACDF) with plating is increasingly performed as the primary treatment of unstable Hangman’s fracture; however, plate-related complications, such as screw back-out, plate fracture and soft-tissue injury, is not uncommon. Polyetheretherketone (PEEK) cage has now been developed to provide initial stability before fusion; however, whether and how ACDF with PEEK cage offer better clinical results compared with ACDF with plating in management of Hangman’s fracture remains unknown. This study compares the efficacy of ACDF with plating to that of ACDF with PEEK cage in management of type II/IIA Hangman’s fractures (according to Levine and Edwards classification) retrospectively.

**Methods:**

From February 2006 to March 2012, a total of 21 patients with type II/IIA Hangman’s fractures combined with intervertebral disc injury underwent ACDF with PEEK cage, and 28 patients underwent ACDF with plating. Perioperative parameters were compared. The average follow-up period was 50.3 months (range 27–76 months). The clinical outcome (visual analog scale (VAS), American Spinal Injury Association (ASIA) scale, and clinical post-traumatic neck score (PTNC)) and radiological outcome (translation of C2, local kyphotic angle (LKA), and fusion status of C2/3) was compared retrospectively.

**Results:**

The operative time and blood loss were significantly less in the ACDF with cage group compared with that in the ACDF with plating group (*P* < 0.05). All patients showed neurological recovery and achieved solid fusion. There were no significant differences in the clinical and radiological outcomes at final follow-up between groups, except in the LKA and the correction loss rate of LKA which were higher in the ACDF with plating group (*P* < 0.05). Donor-site pain occurred in two patients (10.1%) within 6 months after operation in the ACDF with plating group and none in the ACDF with cage group. All patients recovered without any adverse effects.

**Conclusions:**

ACDF with PEEK cage is effective and reliable in management of type II/IIA Hangman’s fractures and is more cost-effective due to shorter operative time and less blood loss requirements.

## Introduction

Traumatic spondylolisthesis of the axis, which accounts for 4%–7% of all cervical fractures/dislocations [1], is the second most common fracture of the second cervical vertebra [2]. It involves a bilateral arch fracture of C2 with a variable degree of displacement of C2 corpus on C3 vertebrae. According to its superficial similarity to the injury seen after judicial hanging due to severe hyperextension and distraction [3], Schneider et al. [4] coined the term “Hangman’s” fracture to describe this injury.

Although the entity of Hangman’s fracture is well known, the optimal strategy of treatment remains controversial [5,6], especially for the type II and IIA fractures combined with disc injury, which are thought to be unstable, according to the classification of Levine and Edwards [7]. In cases of significant displacement and instability, surgical reduction and stabilization is usually preferable [8-10].

Both anterior and posterior approach can be used to treat lesions at C2; however, opinions vary regarding the surgical strategy for treatment of Hangman’s fracture [11]. Although the posterior approach is preferred for its simple exposure, the peculiar anatomy of the upper cervical spine is highly variable, which makes transpedicular screw fixation more technically challenging. Intraoperative neurological and vascular injuries due to misplacement of screws were reported from 11% to 66% of injury rate in its early application [12-14]. The anterior approach, which has the advantage of technical ease and a relatively short fusion, is characterized by anterior C2/3 discectomy and interbody fusion (ACDF) with plating [15]. Some authors have reported good clinical results of this approach in management of Hangman’s fractures, especially for the patients with C2/3 intervertebral disc injury; however, donor site morbidity together with plate-related complications, such as screw back-out, plate fracture, and soft-tissue injury, is not uncommon [16-19].

Polyetheretherketone (PEEK) cage, which became available during the late 1990s, has now been developed to provide initial stability before fusion and widely used in cervical interbody fusion during the past decade [20-22]. Although we have demonstrated the feasibility of PEEK cage solely in management of types II and IIA Hangman’s fracture by biomechanical study [23], to our knowledge, there are few reports on Hangman’s fracture treated by ACDF with PEEK cage solely, especially for those with poor bone quality of vertebral bodies, such as bone cyst, which could not be inserted screws with plating. The purpose of this study was to retrospectively compare the efficacy of ACDF with plating to that of ACDF with PEEK cage solely for treatment of types II and IIA Hangman’s fractures combined with C2/3 disc injuries and then to evaluate the efficacy of ACDF with PEEK solely in management of Hangman’s fractures.

## Materials and methods

### Patients

**Figure 1:**
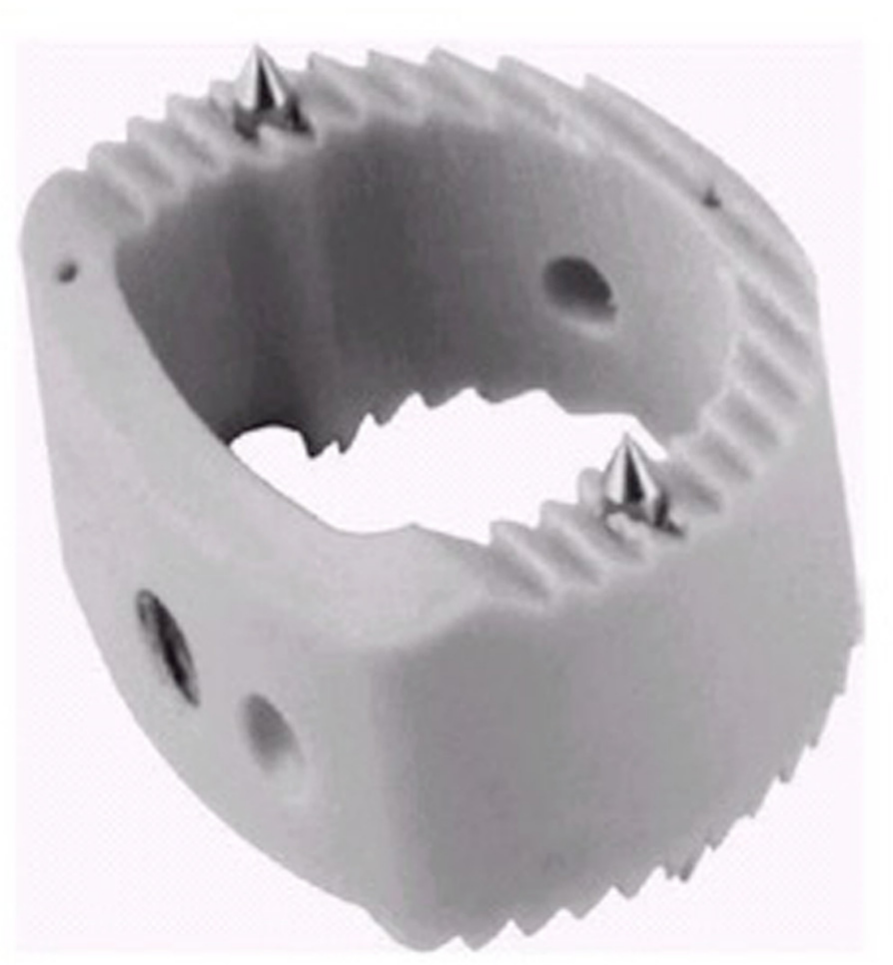
**The image of Solis cage.** It has retention teeth as well as bilateral titanium spikes on the superior and inferior surfaces, which could provide a secure fixation and prevent migration/extrusion of the cage.

From February 2006 to March 2012, a total of 21 patients with unstable Hangman’s fractures who underwent ACDF with PEEK cage (Solis, Stryker Corporation, Cestas, France; Figure 1) and 28 patients who underwent ACDF with plating were included in this study. Inclusion criteria were as follows: types II and IIA (according to the classification of Levine and Edwards) fractures combined with C2/3 disc injury with or without neurologic impairment. There were 30 males and 19 females. Mean age at surgery was 40.9 years (range 26–49 years). The average follow-up period was 50.3 months (range 27–76 months). The follow-up rate was 95.9%. Cases with severe skull injury, metabolic disease, pathological fractures, or combined with multiple fractures of vertebral bodies or extremities were excluded from this study.

Pre- and postoperative neurological status and clinical outcomes as well as that at final follow-up were assessed using the visual analog scale (VAS) form for neck pain, and using the clinical post-traumatic neck score (PTNC) for cervical movement, neurological statue, and daily leaving activities [24]. The recovery rate of PTNC and VAS scores was calculated according to the previous study [25]: the recovery rate of PTNC score = ([PTNC score at final follow up − preoperative PTNV score])/[100 − preoperative PTNC]; the recovery rate of VAS score = ([VAS score at final follow up − preoperative VAS score])/[0 − preoperative VAS score]. The neurological status was graded according to the American Spinal Injury Association (ASIA) scale [26].

**Figure 2:**
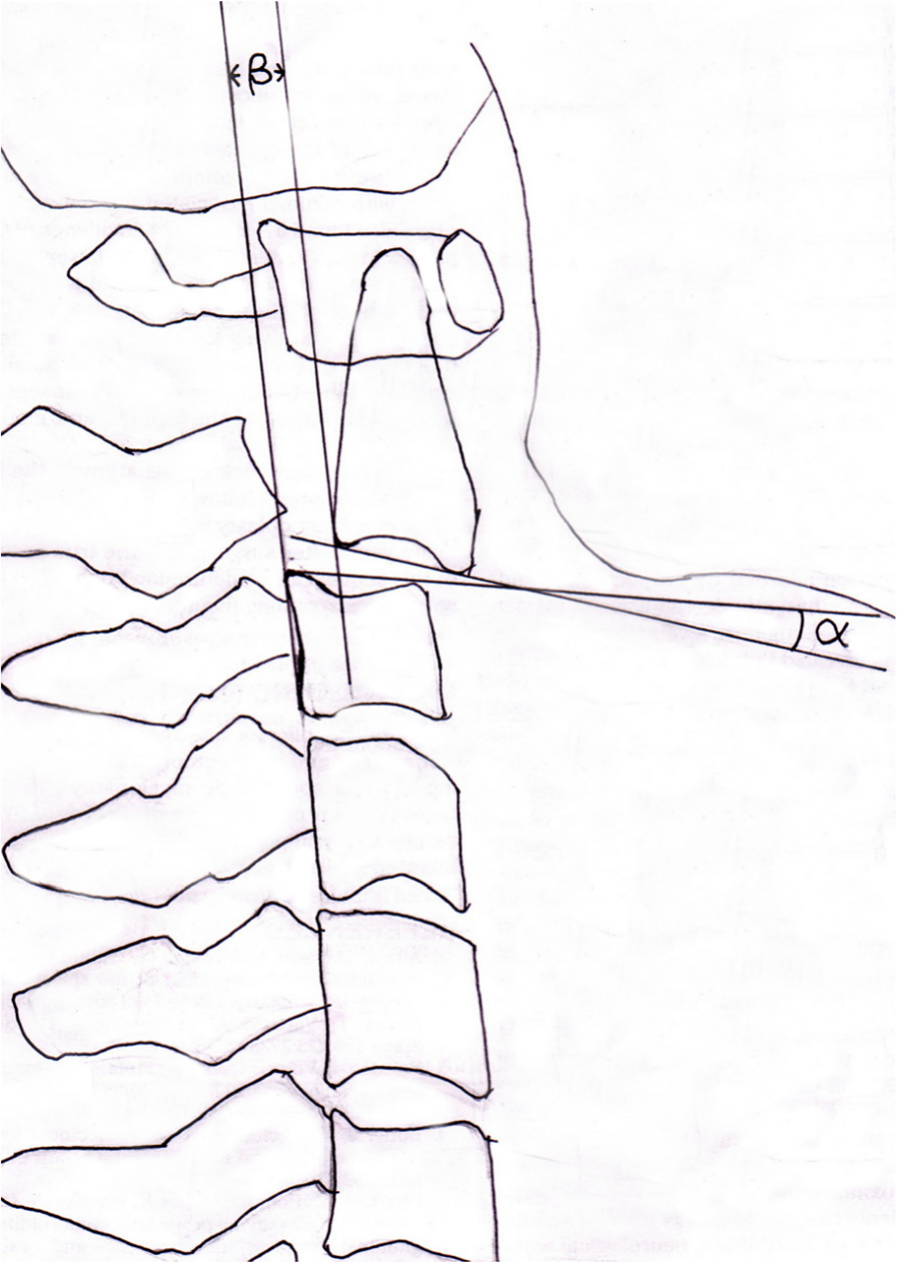
**Diagram showing the local kyphotic angle and translation.** α is the angle between inferior border of C2 and C3. β is the distance between posterior boarders of C2 and C3.

Routine anteroposterior and lateral X-ray films were performed for all the patients before and immediately after surgery, at 3, 6, 12 months, and those with the patient upright at the final follow-up. Local kyphotic angle (LKA) of C2/3 and the anterior translation (AT) of C2 (Figure 2) were measured on X-ray films. The LKA was defined as the angle formed by lines drawn along the inferior endplate of axis and the inferior endplate of C3 [27]. AT was measured as the distance between parallel lines drawn through the posterior border of C3 and the inferior endplate of C2 [28]. Measurements were done on digital radiographs with inbuilt software to measure distance and angles up to the accuracy of 0.01 mm and 0.1° respectively (Philips DICOM Viewer R2.5, Philips Medical Systems Nederland B.V., Best, The Netherlands).The correction of each radiological parameter was calculated by subtracting preoperative parameter from that after operation. The correction loss is the difference between initial postoperative and the parameter at the final follow-up evaluation. The relative percentage of correction loss was calculated as the quotient of the total correction loss over the total correction [29].

The fusion status was also assessed according to the routine flexion-extension radiographs and CT scans for all the patients at 3 and 6 months postoperatively. The criteria of fusion were as follows [30]: (1) trabecular bone across the interfaces and connects superior and inferior vertebral bodies; (2) radiolucency inside the cage disappeared; (3) adequate disc height was restored, without collapse-induced kyphosis; (4) the flexion-extension range of motion at the fusion site was 2° or less.

### Preoperative care and surgical procedure

Skull traction was performed for all the cases preoperatively. According to the type of the individual case, a weight of 3–5 kg with an appropriate angle was applied to stabilize and reduce the fracture. At least a 50% degree of reducing was accomplished in all the cases without advanced neurological deficits or deterioration.

The patient was placed in the supine position with the neck slightly extended and 3–5 kg of axial traction. After anesthesia, fiberoptic bronchoscope-guided nasal intubation was performed. The head of the patient was taped and turned away from side of incision. Surgical procedure was performed using a standard anterior Smith-Robonson approach. A longitudinal incision was made from the angle of the jaw to the hyoid bone. After confirming anatomical position under fluoroscope and C2/3 anterior exposure was obtained, self-retaining retractors were used to facilitate decompression. After elevating the soft tissues cephalad and superiorly, we got enough exposure to access the disc level and to place instruments. Anterior C2/3 discectomy and decompression were performed followed by removing the skull traction after distracting the disc space using the computer-assisted pericardial (CASPER) system under fluoroscopic guidance. The endplates were curetted to remove the cartilage, and the bony endplates were preserved. Under imaging control, the fracture reduction was reduced as much as possible by placing the head in a more slightly extended position and pushing the vertebral body of C2 backward gently to close the remaining gap. Intraoperative sizing was performed for the Solis cage using the templates under fluoroscopic guidance. We used a specialized hollow cylindrical gouge that accompanies the Solis instrumentation set to core a critical amount of cancellous bone out from the iliac crest between the inner and outer tables. The cage was filled with comminuted bone graft and tightly impacted into the prepared disc space, followed by removing the CASPER system. The stability of the cage was routinely checked by anterior drawing actions.

For the patients treated by ACDF with plating, an appropriate tricortical iliac crest was used for interbody fusion, an anterior cervical plate (Johnson and Johnson Professional Inc., Raynham, MA, USA) was selected to allow sufficient purchase on the C2 and C3 vertebral bodies, and final alignment was achieved by tightening the screws. Postoperative immobilization was accomplished with a hard cervical collar for 10–12 weeks for all patients.

### Statistical analysis

The SPSS (version16.0, Chicago, IL, USA) package was used for the statistical analysis. Quantitative data were recorded as the mean and standard deviation. An unpaired Student *t* test, a Mann-Whitney *U* test, and a *χ*^2^ test were used for intergroup comparisons. Intragroup longitudinal comparisons were made using the one-way analysis of variance (one-way ANOVA) and the Friedman’s test, with significance set at *P* < 0.05.

## Results

### Patient demographics

No significant differences were observed between the two groups with respect to age, sex, type of fracture, injury-surgery duration, preoperative VAS and PTNC score, or follow-up period (Tables 1 and 2). In the ACDF with PEEK cage group, the operative time and blood loss was significantly less compared with that in the ACDF with plating group (*P* = 0.003, *P* = 0.011, respectively, Table 3). There was no significant difference in the hospital stay between groups (*P* > 0.05, Table 3).

**Table 1:** **Patients demographics**

	**ACDF + plating (** ***n*** **= 28)**	**ACDF +** **cage (** ***n*** **= 21)**	***P***
Age at surgery (years)	39.2 ± 14.7	42.5 ± 16.1	.812
Gender (male/female)	17/11	13/8	.570
Type of fracture (II/IIA)^a^	18/10	13/8	.615
Duration from injury to surgery (days)	7.5 ± 2.9	8.1 ± 3.2	.650
Follow-up (months)	48.3 ± 29.8	52.1 ± 24.7	.623

^a^According to the classification of Levine and Edwards [7].

*ACDF* anterior C2/3 discectomy and interbody fusion.

**Table 2:** **Clinical outcomes in ACDF with plating versus ACDF with cage group**

	**VAS**			**PTNC**		
	**Preoperative**	**Postoperative (3 ms)**	**FF**	**Preoperative**	**Postoperative (3 ms)**	**FF**
ACDF + plating	6.2 ± 1.1	3.5 ± 1.0	1.8 ± 0.7	35.0 ± 6.5	67.0 ± 10.2	80.1 ± 6.4
ACDF + cage	6.5 ± 1.2	3.0 ± 1.2	1.6 ± 0.5	31.9 ± 7.8	64.6 ± 9.3	82.0 ± 7.7
*P*	.170	.508	.770	.480	.501	.116

*VAS* visual analog scale, *PTNC* post-traumatic neck score, *FF* final follow-up, *ACDF* anterior C2/3 discectomy and interbody fusion.

**Table 3:** **Comparison of surgical parameters**

	**ACDF + plating (** ***n*** **= 28)**	**ACDF + cage (** ***n*** **= 21)**	***P***
Operative time (min)	137.1 ± 37.4	97.4 ± 30.6	.003
Perioperative blood loss (ml)	46.9 ± 16.8	31.3 ± 14.5	.011
Hospital stay (days)	6.9 ± 4.3	6.5 ± 3.8	.783

*ACDF* anterior C2/3 discectomy and interbody fusion.

### Clinical outcome

**Figure 3:**
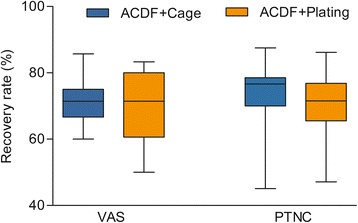
**Comparison of the recovery rate of visual analog scale and post-traumatic neck scores between groups.** *ACDF* anterior C2/3 discectomy and interbody fusion, *VAS* visual analog scale, *PTNC* post-traumatic neck scores.

Both groups showed a significant improvement in VAS score at final follow-up (Table 2), with an improvement of 4.9 points in the ACDF with PEEK cage group (*P* < 0.001) and 4.4 points in the ACDF with plating group (*P* < 0.001). Similar trend was also found in PTNC score, with an improvement of 51.1 points in the ACDF with PEEK cage group (*P* < 0.001) and 45.1 points in the ACDF with plating group (*P* < 0.001, Table 2). The differences between the groups with regard to the recovery rate of VAS and PTNC at the final follow-up were not significant (*P* > 0.05, Figure 3).

No patient deteriorated neurologically during hospitalization and follow-up period. According to the ASIA scale system, 16 patients (76.2%) and 20 patients (71.4%) improved at least one grade at the final follow-up in the ACDF with PEEK cage group and in the ACDF with plating group, respectively. There was no significant difference with regard to the recovery rate of neurological function at the final follow-up between groups (*P* > 0.05).

### Radiological outcome

**Figure 4:**
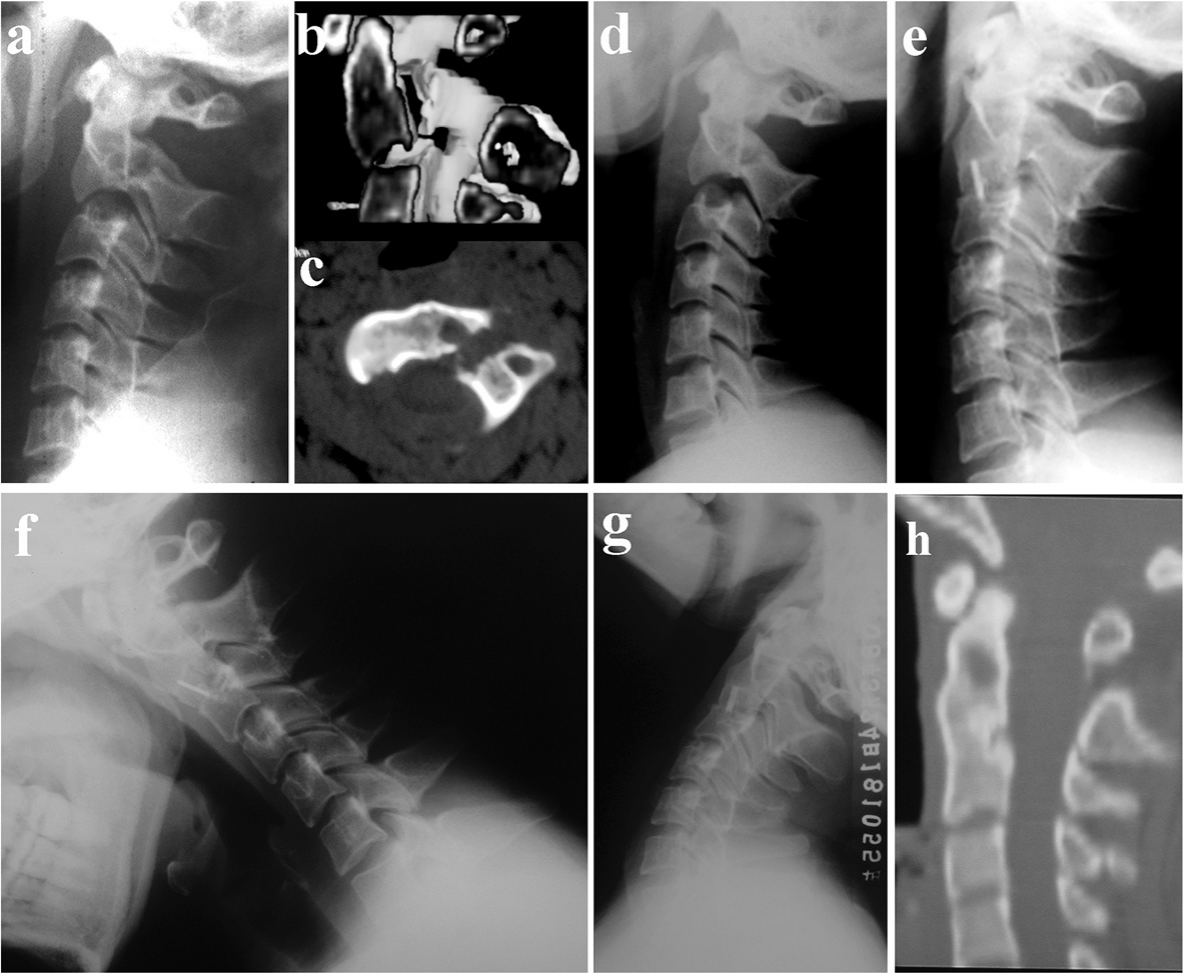
**Images of a 38-year-old male patient. (a, b)** Preoperative lateral X-ray and CT scans showing a type IIA Hangman’s fracture with severe angulation. **(c)** CT with axial section showing a bone cyst in the vertebral body of C2. **(d)** Some degree of reducing was accomplished during skull traction for 3 days. **(e)** Three-month postoperative lateral X-ray after ACDF with PEEK cage showing adequate reduction and bony fusion. **(f, g)** Twenty-four-month flexion/extension lateral X-rays showing no range of motion at the fusion site. **(h)** CT with sagittal reconstruction showing solid fusion and fracture healing. *ACDF* anterior C2/3 discectomy and interbody fusion. *PEEK* polyetheretherketone.

**Figure 5:**
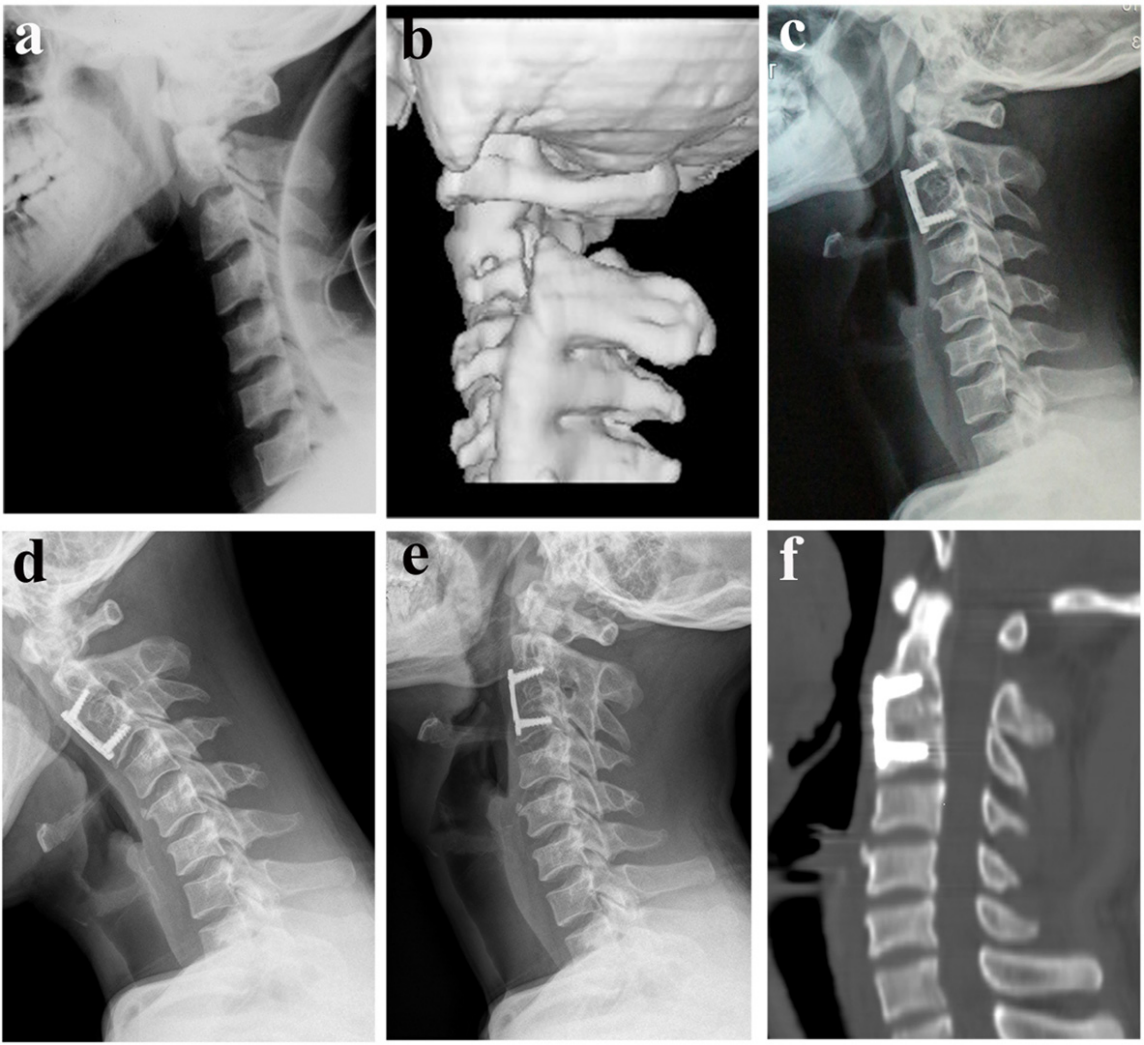
**Images of a 28-year-old male patient. (a, b)** Preoperative lateral X-ray and CT scans showing a type IIA Hangman’s fracture with severe angulation. **(c)** Three-month postoperative lateral X-ray after ACDF with plating showing adequate reduction and bony fusion. **(d, e)** Twenty-four-month flexion/extension lateral X-rays showing no range of motion at the fusion site. **(f)** CT with sagittal reconstruction showing solid fusion and fracture healing. *ACDF* anterior C2/3 discectomy and interbody fusion. *PEEK* polyetheretherketone.

**Figure 6:**
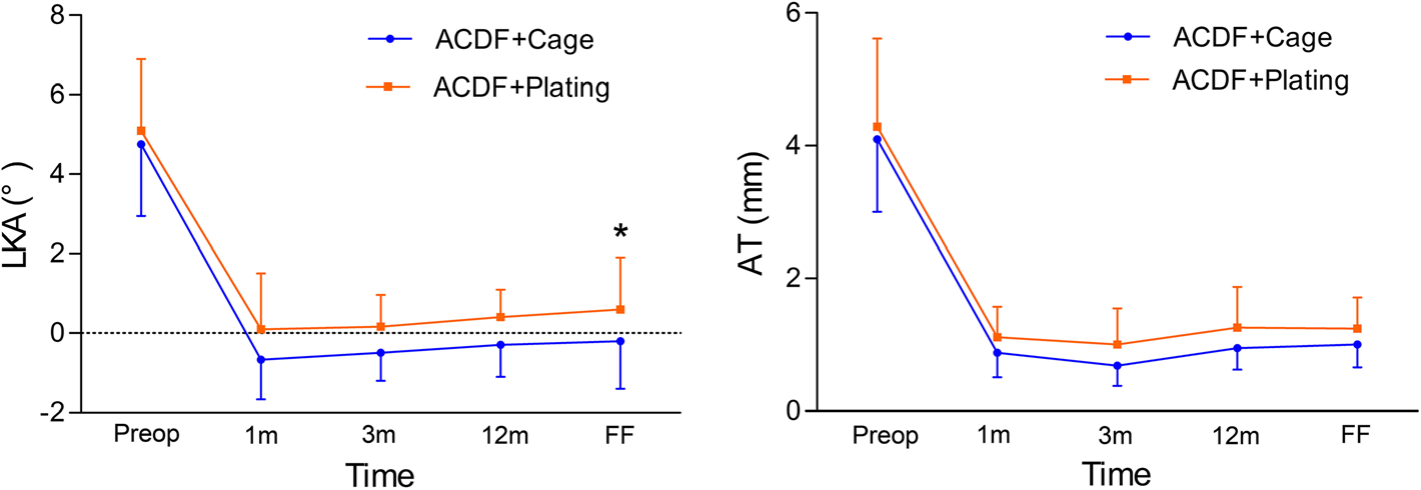
Radiological outcomes based on the local kyphotic angle and anterior translation between groups (* ***P*** **< 0.05).** *ACDF* anterior C2/3 discectomy and interbody fusion, *LKA* local kyphotic angle, *AT* anterior translation.

**Figure 7:**
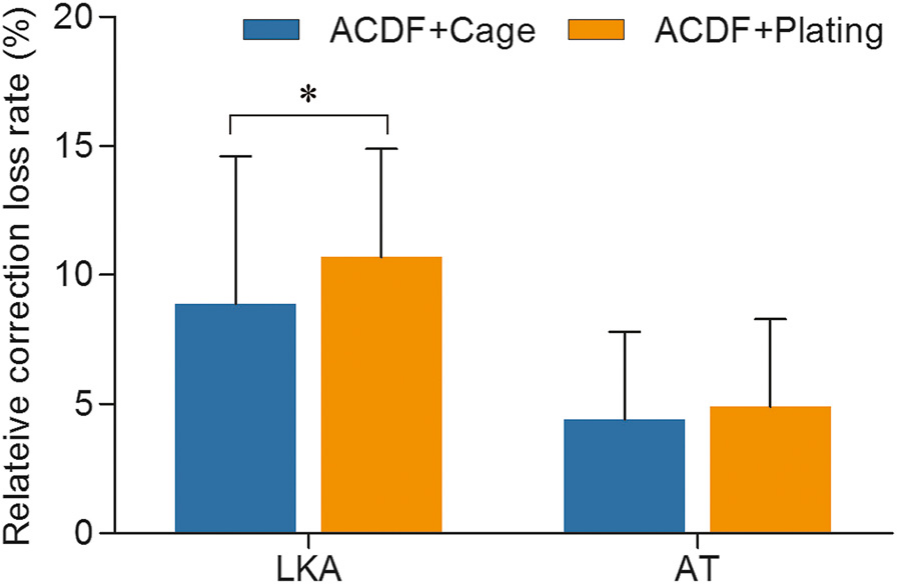
Comparison of the relative correction loss rate of local kyphotic angle and anterior translation between groups (* ***P*** **< 0.05).** *ACDF* anterior C2/3 discectomy and interbody fusion, *LKA* local kyphotic angle, *AT* anterior translation.

All patients showed solid fusion 3 to 6 months postoperatively (Figures 4 and 5). As Figure 5 showed that the sagittal alignment of the fractured segment was satisfactorily restored after surgery as a significant decrease of the LKA and AT was noted in both groups (*P* < 0.001). There was no significant difference in the AT at the final follow-up between groups; however, the LKA at the final follow-up in the ACDF with plating group was significantly higher than that in the ACDF with PEEK cage group (*P* < 0.05, Figure 6). There was no significant difference between the relative correction loss rate of AT at the final follow-up between groups (*P* > 0.05, Figure 7); however, the ACDF with plating group showed a higher relative percentage of correction loss of LKA than the ACDF with PEEK cage group (*P* < 0.05, Figure 7).

### Surgery-related complications

No vascular or neurological complications occurred in either group. In the ACDF with plating group, donor-site pain occurred in two patients (10.1%) within 6 months after operation and in none by 1 year. One approach-related complication, choking and trouble swallowing liquids, was also observed in one patient in the ACDF with plating group and diminished in 3 months with no specific treatment. One superficial infection was found in the ACDF with PEEK cage group and was cured after medication. All patients recovered without any adverse effects.

## Discussion

Although diverse surgical or nonsurgical treatments of Hangman’s fracture have been described, the optimal treatment remains in question [5,6,31]. In the past years, nonsurgical treatments were widely favored in the primary management of a Hangman’s fracture; however, the slow and uncertain healing, as well as the long course of treatment, limited its use. Studies have shown that anterior dislocation, angulation of C2 over C3, pseudarthrosis, and recurrent axial pain occur in about 60% of patients with types II, IIA, and III fractures after conservative treatment [5,7,31]. It was reported that the union rates following conservative management in types II, IIA, and III fractures are 60%, 45%, 35%, respectively [11]. Some authors insisted that nonsurgical management was inappropriate in patients with unstable Hangman’s fractures and discoligamentous injuries, due to the absence of a blood supply to the disc, which is unable to repair itself [32]. This frustrating fact could explain why many surgeons choose primary operation in management of unstable Hangman’s fractures [10,33-35], which could shorten the course of treatment [36].

Surgical stabilization has been accomplished in both anterior and posterior approaches. Due to the complex anatomic feature of the upper cervical spine, the posterior approach was preferred for its relative simple exposure with no major vascular and visceral structure. Among the different posterior approaches, direct posterior fixation of the pedicles or pars fracture with a screw across the fracture line was reported with the advantage of motion reservation in C2-C3 [9,34,37]. However, it had been reported that it was ineffective in management of unstable fractures combined with discoligamentous injury in C2-C3 due to failing of preventing kyphosis and loss of disc height [38]. Redislocations in discoligamentous unstable Hangman’s fracture following direct pars repair have also been reported [6,38]. Although pedicle screw fixation had been reported with good clinical outcomes, it posed the risks of intraoperative neurological and vascular injuries related to screws insertion [12-14]. Yukawa et al. [39] reported that the perforation rate of pedicle screws in C2 and C3 was 21.6%. Although a computer-guided surgical navigation system has been carried out to improve the accuracy of screw insertion [10], these systems were not installed in most hospitals owing to their high cost and user unfriendliness. Another shortcoming of posterior approach was the axial pain after operation, which was not uncommon.

In this instance, some authors advocated an anterior approach for unstable Hangman’s fracture, which has been confirmed to be an effective strategy [5,36,40]. Anterior approach can avoid incorporation of the atlas and thus preserves some rotation movement by sparing the atlanto-axial articulation [41]. Among the anterior approaches, classic ACDF with plating is usually preferred [24,42,43]. Xu et al. [15] reported the results obtained in 28 patients who underwent ACDF with plating for unstable Hangman’s fractures: each patient showed evidence of a solid interbody fusion after 6 months without complications during the follow-up period. Although the utilization of anterior cervical plates helped to achieve good fusion rates, it increased the duration of surgery and was associated with problems of soft-tissue injury as well as instrumentation failure [44]. In addition, anterior cervical plating is inappropriate for patients combined with disease of cervical vertebral body, such as bone cyst, which could not provide sufficient pullout strength at the screw-bone interface.

With the advent of minimal invasive surgery, we utilized ACDF with PEEK cage solely in management of type II/IIA Hangman’s fractures and retrospectively compared the clinical and radiological results with that of ACDF plus plating. Our results showed that both of the two groups achieved 100% bone healing and no significant differences of clinical results were found at final follow-up between groups. A concern arises that ACDF with PEEK cage solely might not provide adequate stability for type IIA Hangman’s fractures. An inconsistent feature of type IIA injuries is that, because of the injury mechanism, the pars interarticularis fractures tend to be more horizontally oriented than in standard type II injuries. So, the prevention of horizontal translocation after trauma deserves more attention. The configuration of the superior and inferior surfaces of the cage conforms to the shape of the respective opposing surfaces of the disc space, and it has retention teeth as well as bilateral titanium spikes on the superior and inferior surfaces, which could provide a secure fixation and prevent migration/extrusion of the cage. In our series, relatively larger implant, which is 1 or 2 mm taller than the original size of C3/4 disc space, was used to ensure the tightness of cage impacted into the disc space, and we routinely checked stability after removal of distracter by anterior drawing of implants in operation, to confirm that the fixation was rigid in each case. We also have performed biomechanical study of this type of cage for type II Hangman’s fracture, which showed that there were no significant differences in range of motion (ROM) of lateral bending and rotation and extension between the cage group and bone graft plus plating group, except in flexion, which could be partly compensated by hard cervical collar [23]. Furthermore, the cage was made of PEEK material, a thermoplastic material with high molecular weight, whose elasticity modulus was similar to that of bone [44]. This helped to minimize stress shielding and subsidence of the cage and allowed optimum interaction of compressive forces at the graft-host interface, which could avoid significant subsidence and correction loss during the follow-up period [44]. As shown in this study, ACDF with PEEK cage group offered lower correction loss rate of local kyphosis in comparison of ACDF with plating group (8.9% for PEEK, 10.8% for plate).

The incidence of donor site morbidity has been reported to be as high as 20% to 30% in some series of ACDF, and deficits included acute and chronic pain, infection, and nerve injury [45-48]. Harvesting of structural corticocancellous autologous bone from the iliac crest may lead to excessive pain and morbidity at the donor site, as well as iliac crest fracture [36,37]. Results from our study were consistent with those in the literature. There were three patients (10.1%) in the plate group complaining donor-site pain until 1 year after operation; however, there were no complications related to the donor site in the cage group. Because there was no need for a structural graft, cancellous bone was harvested via a much smaller opening in the cage group, which could reduce the incidence of morbidity.

In comparison with ACDF with plating procedure, fusion with cage solely is expected to improve upon such variables as duration of hospital stay, operative time, and blood loss. Cauthen et al. [22] reported an even shorter operative time and less blood loss for ACDF with cage procedures, compared favorably with that for plate procedures. Hacker et al. [46] reported an even shorter average hospital stay for cage patients, compared with that for plate patients. Results from our study were largely consistent with those in the literature. Although there was no significant difference in the hospital stay between the plate and cage groups in this study, the operative time and blood loss in the cage groups was significantly less than that in the plate group.

The present study also has some limitations. This is a retrospective study, which is not a randomized control trial or carefully matched comparative cohort study. Therefore, efficacy cannot be validly determined. Furthermore, it was a relatively small-sized study and the number of patients was restricted due to the low incidence of Hangman’s fracture. A multicenter prospective controlled study about these two surgical treatments for Hangman’s fracture should be considered in the future. Nevertheless, we do believe that the anterior approach with the Solis cage stabilization, which can shorten the duration of surgery and medical cost, may be an alternative for unstable Hangman’s fracture when properly indicated.

## Conclusion

This study demonstrated that either ACDF with PEEK cage or with plating could produce satisfactory clinical and radiological outcomes in management of type II/IIA Hangman’s fractures combined with intervertebral disc injury. ACDF with PEEK cage is effective and reliable for the treatment of type II/IIA Hangman’s fractures and is more cost-effective due to shorter operative time and less blood loss requirements.

## Abbreviations

ACDF: Anterior C2/3 discectomy and interbody fusion
PEEK: Polyetheretherketone
VAS: Visual analog scale
PTNC: Post-traumatic neck score
ASIA: American Spinal Injury Association
LKA: Local kyphotic angle
AT: Anterior translation
